# Hematemesis Secondary to Complex Incarcerated Pantaloon Hernia

**DOI:** 10.7759/cureus.13770

**Published:** 2021-03-08

**Authors:** Derrick Huang, Ilya Aleksandrovskiy, Latha Ganti

**Affiliations:** 1 Emergency Medicine, Ocala Regional Medical Center, Ocala, USA; 2 Emergency Medicine, University of Central Florida College of Medicine, Orlando, USA; 3 Emergency Medicine, Envision Physician Services, Plantation, USA; 4 Emergency Medicine, HCA Healthcare Graduate Medical Education Consortium Emergency Medicine Residency Program of Greater Orlando, Orlando, USA

**Keywords:** pantaloon hernia, inguinal hernia, direct hernia, indirect hernia

## Abstract

Hematemesis with concomitant small bowel obstruction is an uncommon emergency department presentation. We report the case of a patient who presented with hematemesis and an incarcerated pantaloon hernia. While the patient initially had intact bowel movements and flatus, he eventually developed complete obstruction that required open surgical repair. In a patient with an incarcerated hernia and a history of recurrent small bowel obstruction, predicting strangulation or compromised bowel and the need for rapid surgical intervention can be difficult. Hematemesis concurrent with hernia incarceration may be suggestive of impending complete bowel obstruction and ischemia.

## Introduction

Hematemesis is a relatively common emergency department (ED) presentation and accounts for 300,000 hospital admissions and approximately 30,000 deaths per year in the United States [1]. This presentation may manifest as either frank red blood or coffee ground emesis and is directly associated with upper gastrointestinal bleeding (UGIB), which is defined as bleeding proximal to the ligament of Treitz, differentiating this source from lower gastrointestinal bleeding (LGIB) that occurs distal to the ligament of Treitz [1]. UGIBs are associated with a six times higher hospitalization rate compared to LGIBs [2]. Etiologically, common causes of UGIB include peptic ulcer disease, gastritis, and esophagitis [3]. However, atypical presentations of hematemesis in the setting of bowel obstruction have been described, such as strangulated bowel obstruction from adhesions and acute volvulus [4,5].

Intraperitoneal adhesions, hernias, and neoplasms account for the vast majority of all small bowel obstructions (SBOs) and have been associated with increasing patient age [6,7]. SBOs are common ED presentations, representing 15% of all ED admissions for abdominal pain [8]. In the setting of bowel obstruction from an incarcerated hernia, ED management is based on risk stratification and hernia reducibility and can range from strangulated bowel requiring emergent surgical intervention to chronic, intermittent obstruction that is amenable to initial nonoperative management [9]. Differentiating between compromised bowel or chronic recurrence, and thus the patient disposition, is made difficult as no clinical, radiological, or laboratory study is completely reliable or free of confounders for diagnosing intestinal ischemia [10,11]. Clues from the history and physical examination may prove beneficial in assessing the need for admission and operative intervention. Our patient presented with coffee ground hematemesis and was found to have an incarcerated, complex pantaloon hernia with compromised bowel.

## Case presentation

An 85-year-old male, with a history of chronic inguinal hernia with remote history of mesh placement, chronic obstructive pulmonary disease (COPD) on 2 L home O_2_, hypertension, and insulin-dependent diabetes presented with hematemesis. Per emergency medical services (EMS) history, the patient was found down and covered with black-colored, coffee ground emesis. The patient had been complaining of abdominal pain and had been weak and less responsive than usual to family members for the last day. The patient endorsed decreased bowel movements. He denied any history of blood thinner or nonsteroidal anti-inflammatory use, alcoholism, or liver cirrhosis.

On his initial vitals, the patient had a pulse oximetry of 98% on 2 L of nasal cannula, a blood pressure of 123/72 mmHg, temperature of 36.7°C, heart rate of 115 beats per minute, and a respiratory rate of 18 breaths per minute. On physical examination, the patient was alert and oriented without distress, with coffee ground emesis on his clothes. His head and body were without obvious external signs of trauma. A cervical collar was placed by EMS. The patient was tachycardic with a regular rhythm, equal pulses bilaterally, and lungs were clear to auscultation. Abdomen was greatly distended with localized tenderness to the right lower quadrant without guarding or rigidity. A nonreducible, tender, right-sided hernia with extension into the scrotum was found on groin examination. Skin examination was unremarkable.

**Figure 1:**
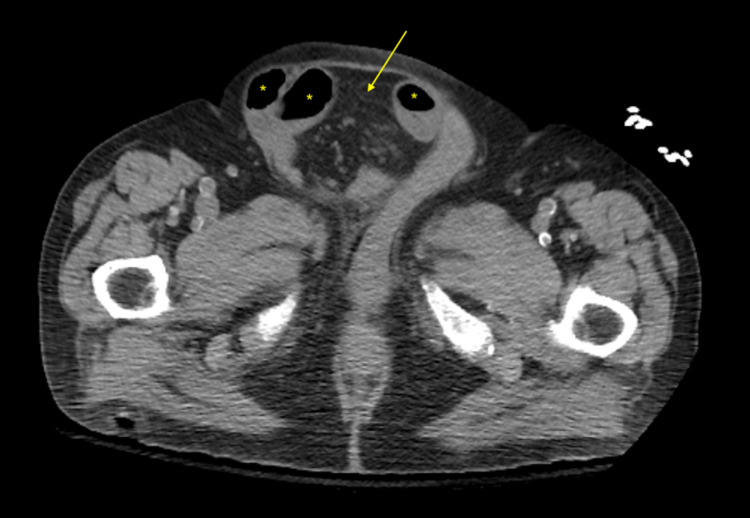
CT scan demonstrating a large right-side indirect hernia (arrow) with multiple loops of small bowel extending into the hernia defect (asterix), along with a direct hernia and small bowel distention throughout the abdomen. CT, computed tomography

Initial laboratory studies were remarkable for a blood urea nitrogen (BUN) of 105 mg/dL and creatinine 2.7 mg/dL, with a BUN/creatinine ratio (BUN/Cr) of 38.9 and a lactic acid of 3.4 mmol/L. Glucose was 672 mg/dL. Hemoglobin and hematocrit were within normal ranges. Troponin and electrocardiogram were unremarkable. White blood cell count, lipase, creatine kinase, liver function tests, and electrolytes were also unremarkable. Computed tomography (CT) scan of the head and neck without contrast were unremarkable. Chest radiography showed a right upper lobe pulmonary infiltrate. CT scan of the abdomen with contrast showed an SBO caused by a large right-side indirect hernia with multiple loops of small bowel extending into the hernia defect, along with a direct hernia and small bowel distention throughout the abdomen (Figure 1).

**Figure 2:**
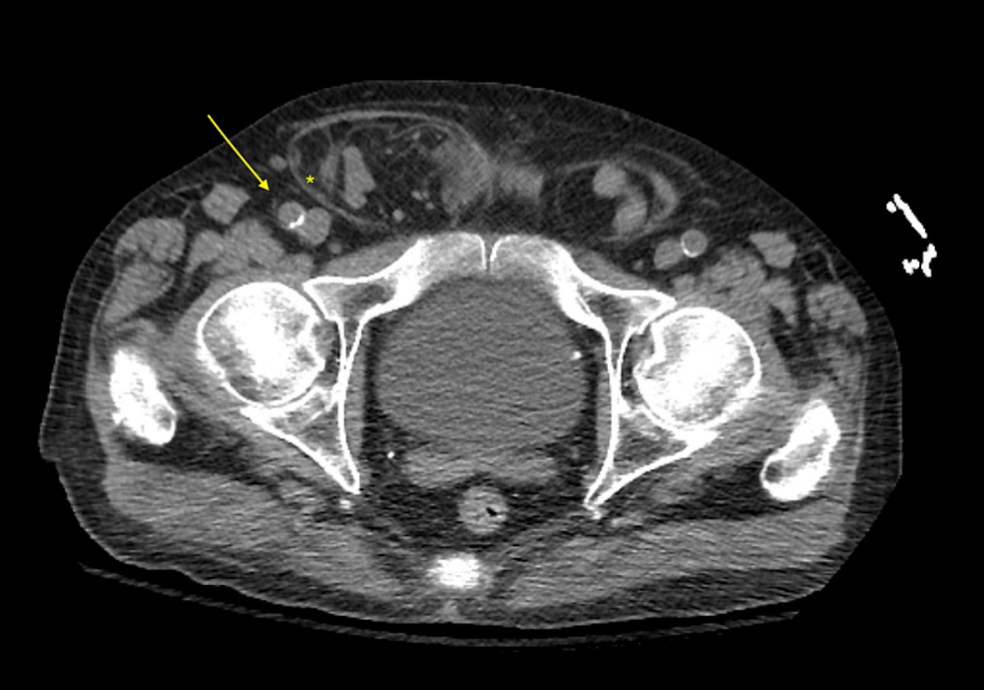
CT scan demonstrating the small bowel extending all the way into the right hemiscrotum through the right inguinal canal. CT, computed tomography

The small bowel extended all the way into the right hemiscrotum through the right inguinal canal (Figure 2).

The patient was seated upright, given intravenous fluids, pantoprazole, ondansetron, and analgesia. Blood cultures were obtained before administration of ceftriaxone. Given laboratory and imaging findings, worsening symptoms, and clinical presentation, the patient was admitted. During admission, he became acutely obstructed without flatus and bowel movement, and he ultimately underwent right open inguinal hernia repair, where he was found to have massive incarcerated right pantaloon inguinal hernia, extensive adhesions, and a meshoma from a previous inguinal surgery. A pantaloon hernia consists of concurrent direct and indirect inguinal herniae where hernial sacs are present on both sides of the inferior epigastric vessels and are separated by the posterior wall of the inguinal canal. There were multiple loops of small bowel, some with adhesions to the hernia sac at the level of the scrotal component requiring right pantaloon hernia repair with mesh, right orchiectomy, and extensive lysis of adhesions. The patient was sent to the intensive care unit post-operatively in a stable condition and he was ultimately discharged without further complications.

## Discussion

We report a case of hematemesis with concomitant incarcerated complex hernia ultimately requiring open surgical repair during admission. Our patient was first assessed for the severity of his UGIB and syncope. He was given ceftriaxone for pneumonia given his chest radiograph findings and empiric prophylaxis for superimposed bacterial infection in the setting of hemorrhage and possible liver cirrhosis [12]. The patient was deemed stable based on an intact ability to protect his airway, stable vital signs, and an unremarkable troponin and electrocardiogram. Given the complex patient presentation, CT abdomen and pelvis findings, and his incarcerated hernia, the patient was admitted for observation and the potential need for surgical repair. Our patient’s coffee ground emesis presentation along with the substantially elevated BUN/Cr of 38.9 ostensibly was evidence of an “upper” GI bleed atypically presenting in the context of an incarcerated hernia. BUN/Cr greater than 30 has been shown to suggest a UGIB with a positive likelihood ratio of 7.5 [13]. Although the source of hematemesis is typically associated with a bleeding source above the ligament of Treitz, obstructions have been described with hematemesis [4,5]. Obstructed bleeding resulting from compromised, potentially ischemic lower GI bowel may be more susceptible to digestion, resulting in substantially elevated BUN as well as the coffee ground quality, which may suggest a slower or more distal bleeding process compared to frank red blood [14]. Of note, the hemoglobin and hematocrit may remain normal initially, given that the patient has lost whole blood. SBOs are also associated with dehydration, which may be a confounder contributing to the elevated lactic acid, as well as the pre-renal acute kidney injury from the elevated BUN/Cr; however, the ratio remained elevated after adequate fluid resuscitation.

Indirect hernias are the most common groin hernia in both males and females [15]. Most indirect hernias in adults are congenital, resulting from a dysfunctional shutter mechanism in patients with a patent processus vaginalis [16]. Increases in intra-abdominal pressure, as seen in chronic coughing from COPD as in our patient, may force abdominal contents thought the internal ring into the inguinal canal, resulting in herniation. Additionally, as in our patient, atypical presentations are possible as exemplified by a pantaloon hernia wherein both indirect and direct hernia present simultaneously, further complicating the case. Risk factors for incarceration and strangulation include old age, femoral hernia, and recurrent hernia [17]. Our patient’s advanced age and history of recurrent hernia both increased his risk for incarceration and required emergent surgical intervention.

In the ED, the primary concern in evaluating a hernia is assessing for strangulation requiring emergent surgical intervention, followed by intravenous fluid resuscitation, controlling emesis, and analgesia. A nasogastric tube may be symptomatically beneficial for patients with substantial distension and vomiting by extracting contents proximal to the site of obstruction. Clues for strangulation may include symptoms of bowel obstruction, such as nausea, vomiting, abdominal pain, and distention, as well as systemic symptoms if strangulation and ischemia have occurred, along with abdominal tenderness and elevation in lactic acid. Most SBOs resolve from nonoperative management, and chronically incarcerated hernias (as in our patient) have been known to have success with nonoperative management and may be independently associated with a reduced likelihood of requiring operative intervention at any point during a hospitalization [9,18]. However, this is balanced by patient age and comorbidities and the risk that a surgical delay of even one day may lead to longer and more complicated post-operative hospital stays [19]. Although our patient had a history of recurrent, chronic bowel obstruction, recent bowel movements and flatus, and relatively unremarkable laboratory findings, atypical clinical clues such as hematemesis in the context of an incarcerated hernia prompted immediate admission and surgical consultation. Our patient subsequently developed complete obstruction during admission and received prompt surgical repair without delay.

## Conclusions

In the atypical context of hematemesis and signs of obstruction, ED management centers on stabilization of the patient, assessing for a potentially life-threatening UGIB, and assessment for bowel strangulation requiring emergent surgical intervention. Our patient was ultimately diagnosed with an acute on chronic SBO resulting from a complex, incarcerated pantaloon hernia and received prompt surgical care. Hematemesis occurring in the context of an incarcerated hernia may be a marker for hernia complexity and threatened bowel with impending obstruction.
